# Imbalanced ECG signal-based heart disease classification using ensemble machine learning technique

**DOI:** 10.3389/fdata.2022.1021518

**Published:** 2022-10-10

**Authors:** Adyasha Rath, Debahuti Mishra, Ganapati Panda

**Affiliations:** ^1^Department of Computer Science and Engineering, Siksha O Anusandhan (Deemed to be) University, Bhubaneswar, Odisha, India; ^2^Department of Electronics and Tele Communication, C. V. Raman Global University, Bhubaneswar, Odisha, India

**Keywords:** ensemble model-based HD detection, classification of HD using imbalanced ECG records, SVM, AdaBoost, LR

## Abstract

The machine learning (ML)-based classification models are widely utilized for the automated detection of heart diseases (HDs) using various physiological signals such as electrocardiogram (ECG), magnetocardiography (MCG), heart sound (HS), and impedance cardiography (ICG) signals. However, ECG-based HD identification is the most common one used by clinicians. In the current investigation, the ECG records or subjects have been sampled and are used as inputs to the classification model to distinguish between normal and abnormal patients. The study has employed an imbalanced number of ECG samples for training the various classification models. Few ML methods such as support vector machine (SVM), logistic regression (LR), and adaptive boosting (AdaBoost) which have been rarely used for HD detection have been selected. The performance of the developed model has been evaluated in terms of accuracy, F1-score, and area under curve (AUC) values using ECG signals of subjects given in publicly available (PTB-ECG, MIT-BIH) datasets. Ranking of the models has been assigned based on these performance metrics and it is found that the AdaBoost and LR classifiers stand in first and second positions. These two models have been ensembled based on the majority voting principle and the performance measure of this ensemble model has also been determined. It is, in general, observed that the proposed ensemble model demonstrates the best HD detection performance of 0.946, 0.949, and 0.951 for the PTB-ECG dataset and 0.921, 0.926, and 0.950 for the MIT-BIH dataset in terms of accuracy, F1-score, and AUC, respectively. The proposed methodology can also be employed for the classification of HD using ICG, MCG, and HS signals as inputs. Further, the proposed methodology can also be applied to the detection of other diseases.

## Introduction

Cardiovascular disease (CVD) is a generalized term that includes diseases relating to the heart as well as blood vessels (Anooj, 2012). The various types of CVDs are: coronary artery disease (CAD), VHD, heart failure (HF), coronary heart disease (CHD), peripheral artery disease, and angina (Anooj, 2012; Dwivedi, 2018). These variants of CHDs are diagnosed either by clinical test data, ECG, HS, echocardiography, ICG, or MCG signal (Dwivedi, 2018; Kumar and Gandhi, 2018). It is observed that the ECG is mostly used by physicians for detecting the HD of a subject. The classification / detection of CVD mostly employs soft computing, evolutionary computing, ML as well as deep learning (DL)-based approaches. In this section, a detailed review of the existing literature on CVD detection is presented.

In Anooj (2012), the authors have developed a clinical decision support system for HD risk prediction from the clinical test data using the fuzzy logic technique. The experimental results using the University of California, Irvine (UCI) repository show that the proposed method outperforms neural network-based classifiers in terms of accuracy, sensitivity, and specificity. In an interesting article (Dwivedi, 2018), the author investigated HD prediction using different ML techniques. It is reported that the LR classifier provides highest accuracy, sensitivity, and specificity of 85, 89, and 81%, respectively. A non-invasive internet of things (IoT) platform-based HD detection scheme has been proposed in Kumar and Gandhi (2018) by employing clinical data. The proposed scheme involves a three-tier IoT architecture. The author has also made a receiver operating characteristic (ROC) analysis to find the significant clinical parameters responsible for detection. The random forest (RF), as well as hidden Markov model (HMM)-based HD classification models, have been suggested in Meng et al. (2019) by employing activity tracker data. It is found that the HMM model provides higher AUC of 0.79 compared to that (0.76) provided by the RF model. For the prediction of HD, a hybrid scheme (Mohan et al., 2019) using the linear model (LM) and RF has been developed under the IoT platform. It is shown that the proposed hybrid scheme yields an accuracy of 88.7%. For the diagnosis of CAD, the binary-real particle swarm optimization (PSO)-based hybrid scheme using two different feature selection methods has been employed in Zomorodi-moghadam et al. (2021). It is observed that the selected 11 features outperform the classification results compared to the 13 feature-based models. A novel approach to HD prediction has been reported (Magesh and Swarnalatha, 2021) by using the cluster-based decision tree (DT) and RF classifier from UCI repository data. The suggested approach provides higher classification accuracy of 89.3% compared to 76.70% accuracy yielded by the same classifier without cluster-based DT learning. In an interesting article (Li et al., 2020), five different ML-based HD identification models have been reported. The classifiers used in these methods are k-nearest neighbor (k-NN), DT, LR, and artificial neural network (ANN). The authors have introduced a fast conditional mutual information-based feature selection approach (FCMIM). In addition, other feature selection algorithms such as relief, least absolute shrinkage selection operator (LASSO), minimal redundancy maximal relevance (MRMR), and local learning-based methods have been employed for comparing the performance measures. It is reported that the proposed FCMIM-based support vector machine (SVM) classifier produces highest accuracy of classification. Using the clinical test data, a non-invasive CHD detection method is proposed in Wang J. et al. (2020a). This method employs base and meta-level stacking. It is reported that the suggested scheme provides specificity, sensitivity, and accuracy of 94.44, 95.84, and 95.43%, respectively. The ML techniques such as k-NN, NB, and binary logistics have been used to develop the individual as well as ensemble models using the principle of bagging, boosting, and stacking for the detection of CHD from clinical data (Shorewala, 2021). The boosted models provide highest AUC score of 0.73. But the stacked model is found to be the best with an accuracy of 75.1%. In another work (Oresko et al., 2010), the authors have proposed a real-time CVD detection method from the ECG sample. It can be implemented in a smartphone-based platform. A long short-term memory (LSTM) network has been trained (Ganguly et al., 2020) using ECG signal for the automatic classification of arrhythmia. It is shown that a bi-directional LSTM (b-LSTM) network outperforms another LSTM model. The CHD risk detection using ECG samples has been achieved under a mobile cloud computing environment (Venkatesan et al., 2018). The proposed method has employed wavelet transform (WT) for the detection of R-peaks. The adaptive neuro-fuzzy inference system (ANFIS) approach has been followed to develop as a classifier. A hybrid approach using WT and b-LSTM has been employed for the classification of ECG signal (Yildirim, 2018). It is shown that the proposed model provides a recognition performance of 99.39%. A CVD classifier employing ECG signal has been developed (Deng et al., 2018) following the dynamical neural learning mechanism. The effectiveness of the proposed scheme has been proved using (PTB-ECG datasets, (2004)). A modified RF along with an improved LM for detecting HD on the internet of medical things platform (IoMT) has been developed in Guo et al. (2020). The proposed scheme provides 96.6, 96.8, and 96.7% of accuracy, stability ratio, and F1-score, respectively. An automated convolutional neural network (CNN)-based heartbeat classifier has been developed (Wang H. et al., 2020c) using ECG records and its various performance measures have been evaluated. It is reported that the suggested model detects arrhythmia with an accuracy of 99.06%. In another article (Hussain et al., 2020), the authors have developed a model to detect HF. To achieve this, they have employed SVM, DT, k-NN, and ensemble classification models and multi-dimensional features. It is observed that the SVM classifier provides a sensitivity of 96%, specificity of 89%, and accuracy of 93.1%. The HD has been diagnosed using deep learning neural network (DLNN) and CNN-based models (Rath et al., 2021a). It is found that the accuracy of classification, sensitivity, and specificity varies between 89–99, 91–97.5, and 92.83–99.2%, respectively. Most of the ML and DL models provide satisfactory CVD detection from balanced ECG samples. However, in Rath et al. (2021c), the authors have suggested generative adversarial network (GAN) and LSTM models to detect CHD from two types of imbalanced datasets. It is shown that the GAN model outperforms all other models but the GAN-LSTM ensemble model provides the best CHD detection performance from the imbalanced datasets. In another interesting article (Sengur and Turkoglu, 2008), an artificial immune system-based fuzzy k-NN classifier has been suggested to detect heart valve disorders using Doppler HSs. It is reported that the proposed method yields 95.9 and 96% sensitivity and specificity rates, respectively. The incremental self-organizing map (ISOM) as well as Kohonen's SOM have been used as classifiers of HS (Dokur and Ölmez, 2008). The WT has been employed for segmentation as well as for the extraction of features. It is found that the ISOM model satisfactorily classifies the HS in the noisy environment. A radial wavelet neural network (RWNN) with an extended Kalman filter (EKF)-based training scheme has been used (Guillermo et al., 2015) as a classifier for detecting the heart murmur. The results of this model have been compared with an ANN model using Levenberg–Marquardt training. The authors in Liu et al. (2019) have developed an extreme learning machine (ELM) classifier for the identification of HF from the characteristics of HS. They have used 11 features extracted from the HS. The proposed method provides 96.32, 95.48, and 97.10% accuracy, sensitivity, and specificity, respectively. The SVM classifier has been used (Abduh et al., 2020) for classifying HS using mel-frequency spectral coefficients. It is shown that the proposed scheme offers a sensitivity of 0.8735 and specificity of 0.9666. The detection of HD from the HS signal of children has been obtained by employing an ANN classifier. The HS has been segmented using discrete wavelet transform (DWT) as well as the Hadamard product (Wang J. et al., 2020b). It is observed that the detection accuracy, specificity, and sensitivity of 93, 91.7, and 93.5%, respectively, have been achieved by the proposed model. Very few works have been carried out on HD detection employing MCG signal. In Tao et al. (2018), the authors have employed the SVM-extreme gradient boost (XGBoost) hybrid model providing the best performance metrics compared to other methods. Three different classifiers (DT, RF, and SVM) have been chosen to diagnose (Salah et al., 2020) the VHD from the ICG signal. The authors have extracted the statistical, morphological, and spectral features from the ICG samples. Subsequently, principal component analysis (PCA) has been used to reduce the number of features. It is observed that the combination of these three features-based RF classifiers provides highest accuracy of 96.34%. Many DL-based classifiers have been employed for the detection of CVD from mammograms (Wang J. et al., 2017). A 12-layer CNN has been trained to identify breast arterial calcification (BAC). It is observed that the proposed approach achieves a detection efficiency similar to human experts. A critical review article (Rath et al., 2021b) has been reported on the diagnosis of HD using various clinical data, ECG, and HS samples. It also presents various types of datasets, different feature extraction and reduction techniques, and various ML and DL classifiers for HD detection.

The analysis of the literature review reveals that many standard ML methods have already been used for CVD detection from ECG signal of subjects. However, it is observed that many ML methods such as AdaBoost (Wang J. et al., 2020a) and LR (Dwivedi, 2018) have been employed as a classifier in a few cases. Further, the validation task of the detection model has been carried out using only one source of standard ECG samples (Oresko et al., 2010; Ganguly et al., 2020). Third, in few articles, the ensemble model has been suggested (Hussain et al., 2020; Shorewala, 2021) using the ML models for achieving enhanced detection performance. In most of the articles, the training and validation operations of the ML and DL models have been carried out using a balanced number of ECG signals of subjects. These observations have encouraged developing of ML-based detection models using LR and AdaBoost classifiers. Further, to assess the consistent performance of the proposed models, the standard MIT-BIH and PTB-ECG-based ECG datasets have been chosen both during the training and validation phases.

The imbalanced data mean the number of normal and abnormal patients is not equal. When the number of normal and abnormal cases is not equal, the model is trained with a bias toward higher number of the two classes. The model which is developed under such conditions provides a lower accuracy of detection. So, the challenge is to achieve improved training and testing results under the imbalanced condition of the input data.

Many ML methods exhibit poor detection performance when the training and testing datasets are imbalanced. Therefore, in this article, imbalanced ECG samples have been employed to examine the performance potentiality of the classifier. With an objective to further improve the detection accuracy, an ensemble model has been developed by choosing the best of the three ML classifiers.

Based on the motivation and objectives of the proposed work, the article has been organized in the following way. Section “Materials and mehods” deals with the materials and methodology required for CHD detection from imbalanced ECG samples. It provides the details of the standard data sources used as well as the training and testing schemes of SVM, LR and AdaBoost and ensemble version of LR and AdaBoost classifiers. Section “Simulation based experiments” outlines the simulation-based experiments obtained using the trained models of Section “Materials and methods.” The analysis and discussions on various results have also been made in Section “Discussions.” It also presents the contribution of the article. Finally, Section “Conclusion” provides the concluding remarks of the investigation and suggests the scopes of future research work.

## Materials and methods

This section presents the details of materials in terms of ECG recordings of normal and abnormal subjects available from two standard ECG datasets. The block diagram/flowchart of three classifiers and the corresponding training and testing steps are provided in this section.

### Materials

The two datasets which are used for obtaining ECG samples are MIT-BIH and PTB-ECG (Bousseljot et al., 1995; Goldberger et al., 2000; George Moody and Mark Roger, 2001). The details of these two datasets are available in MIT-BIH ECG datasets, (2005) and PTB-ECG datasets, (2004). The details of the numbers of normal and abnormal cases and numbers of training (70%) and testing sets (30%) are shown in Table 1. As evident from this table, the number of normal and abnormal cases chosen is imbalanced.

**Table 1:** Materials used for training and testing the ML classification models.

**Datasets**	**No. of ECG datasets**	**No. of abnormal cases**	**No. of normal cases**	**Training sets (70%)**	**Testing sets (30%)**
MIT-BIH	268	104	164	188 normal-115 abnormal-73	80 normal-49 abnormal-31
PTB-ECG	200	54	146	140 normal-104 abnormal-36	60 normal-42 abnormal-18

### Methodology

From each subject, 12 ECG recordings have been taken and averaged to achieve a smooth ECG waveform. The average ECG waveform of each subject has been sampled to produce 1,024 discrete samples. At a time, all the samples of a subject have been fed to each classification model for training and validation purposes. In this study, the 1,024 samples of each ECG signal are considered. The 1,024-dimensional sample vectors for the ECG signals are used for the training and testing of the classifiers for HD detection. Four classification models such as logistic regression (LR), SVM, AdaBoost, and LR-AdaBoost are used in this work.

### Logistic regression

It is a predictive classification algorithm that assigns a class to the set of measurements or observations (Scott, 2002). It employs a sigmoid function to limit the output between 0 and 1. The output of the LR equation is computed as


(1) $$\begin{array}{l} z\;=\;\alpha_{0}+\alpha_{1}\left(x\;\right) \end{array}$$

(2) $$\begin{array}{l} sig\left(z\right)=\;\frac{1}{1+e^{-z}} \end{array}$$

(3) $$\begin{array}{l} {\;f}_{\theta }\left(x\right)=sig\left(z\;\right) \end{array}$$

The cost function is given by (Scott, 2002).


(4) $$J\left(\theta \right)=\frac{1}{2}\sum {}_{k=1}^{K}{\left(f_{\theta }\left(x^{\left(k\right)}\right)-y^{\left(k\right)}\right)}^{2}$$

For a two-class problem, *y is equal to either* 1 *or* 0. The cost function is minimized with respect to θ, to obtain the update equation (Scott, 2002).


(5) $$\begin{array}{l} \theta_{j}=\;\theta_{j}-\alpha \left(f_{\theta }\left(x\right)-y\right)x \end{array}$$

The symbol α denotes the learning rate which lies between 0 and 1 and needs to be suitably adjusted during the training phase. After the completion of the training, the performance metrics of the model are evaluated.

### Support vector machine

The principle of the SVM classifier is explained in steps. Let *X* represents the samples of ECG recording of subjects and *Y* represents the corresponding class vector (Cortes and Vladimir, 1995). The key steps of SVM classifier during the training phase are:

Step 1: Compute *Y*^*T*^*Y* and *XX*^*T*^
Step 2: Compute the matrix *H* = *Y*^*T*^*Y*.*X*^*T*^*X*
Step 3: Compute the Lagrangian Multipliers, α.
Step 4: Compute decision hyperplane normal vector, *W* = (α.*Y*)^*T*^.*X*
Step 5: Compute bias, $b=1-w^{T}x_{1}$

During the testing phase, the class of unknown ECG samples, *z* is evaluated by computing *sign*(*w*^*T*^*z*+*b*). If it is positive, then the test dataset belongs to class 1 (Cortes and Vladimir, 1995).

#### AdaBoost

The AdaBoost algorithm is an ensemble method of ML (Schapire, 2013). In this case, higher weights are assigned to wrongly classify instances. The boosting is used to minimize the bias and the variance for supervised learning. Excluding the first one, each subsequent learner is developed from the previous ones. The AdaBoost is based on the principle that weak learners are transformed into strong ones. The block diagram of the AdaBoost-based classifier is shown in Figure 3. The ECG samples of subjects are fed to the first model (in this case DT). In this case, the first model is built and the errors from this model are noted. The ECG record which is incorrectly classified is fed to the next model (Schapire, 2013). This process is continued until a pre-specified condition is made. In this case, the algorithm only makes a node with two leaves which is called a stump. The major steps of building the classifier are

Step 1: To create the first base learner by taking the first feature and the process is continued for all features. So, the number of base learners or stumps is equal to the number of features.
Step 2: To calculate the total error is *E* = 1/*N*, where *N* is equal to the number of records.
Step 3: To compute the performance (*P*) of the stump according to

$$\begin{array}{l} P=\frac{1}{2}\ln\frac{\left(1-E\right)}{E}\; \end{array}$$

where, ln denotes the natural log.

Step 4: To update the sample weights according to

$$\begin{array}{l} New\;Weight=Old\;weight\;\times e^{-\left(Performance\right)}\; \end{array}$$

where, the initial weight = $\frac{1}{N}$

Step 5: To create a new dataset by choosing incorrectly classified records as well as a few correct ones.
Step 6: To create a set of new DTs (stump) and continue the process until the last error is produced.

#### LR-AdaBoost ensemble model

So, in the present case, the ML techniques are primarily used to develop prediction or classification tasks. Each of the developed model provides the accuracy of classification based on their potentiality. To further improve the accuracy of performance, the ensemble model is developed using each of the basic model. In this process, the outcome of the overall ensemble model becomes better than the individual model which is part of the combination model. The challenging case of the ensemble model is to determine the connecting weights of each individual model. Mostly this is achieved by majority voting or bio-inspired-based optimization techniques.

To improve the classification performance, the ensemble model is developed by choosing the two best models (Polikar, 2006). In the present case, the LR and AdaBoost models are first trained and these pre-trained models are connected in parallel as shown in Figure 4. The input to the ensemble scheme is the samples of each record of the standard ECG dataset. The output of each of these models is fed to the majority voting scheme. The final predicted class (normal/abnormal) refers to the output of the majority voting scheme (Polikar, 2006). This principle classifies the input records in a superior way compared to each individual model.

The various results obtained from the simulation study of the three ML and one ensemble models have been obtained and have been tabulated and plotted in the next section.

## Simulation-based experiments

**Figure 1:**
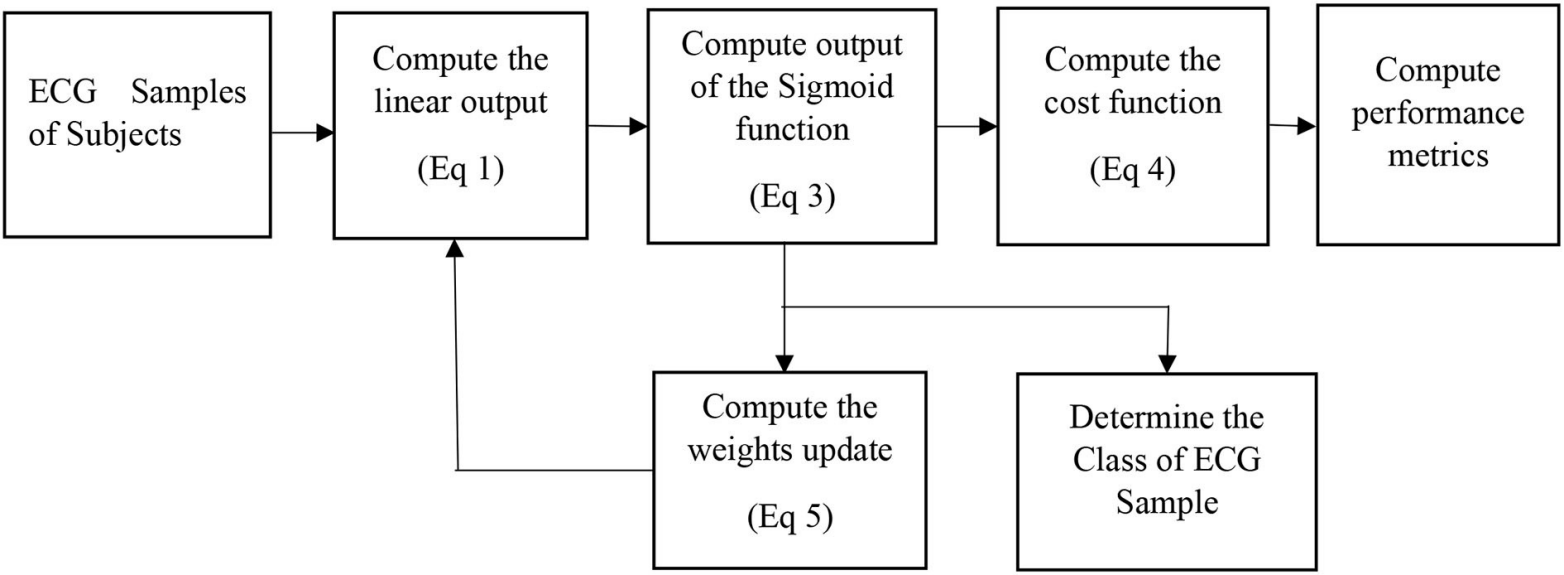
Block diagram of the logistic regression-based classification model.

**Figure 2:**
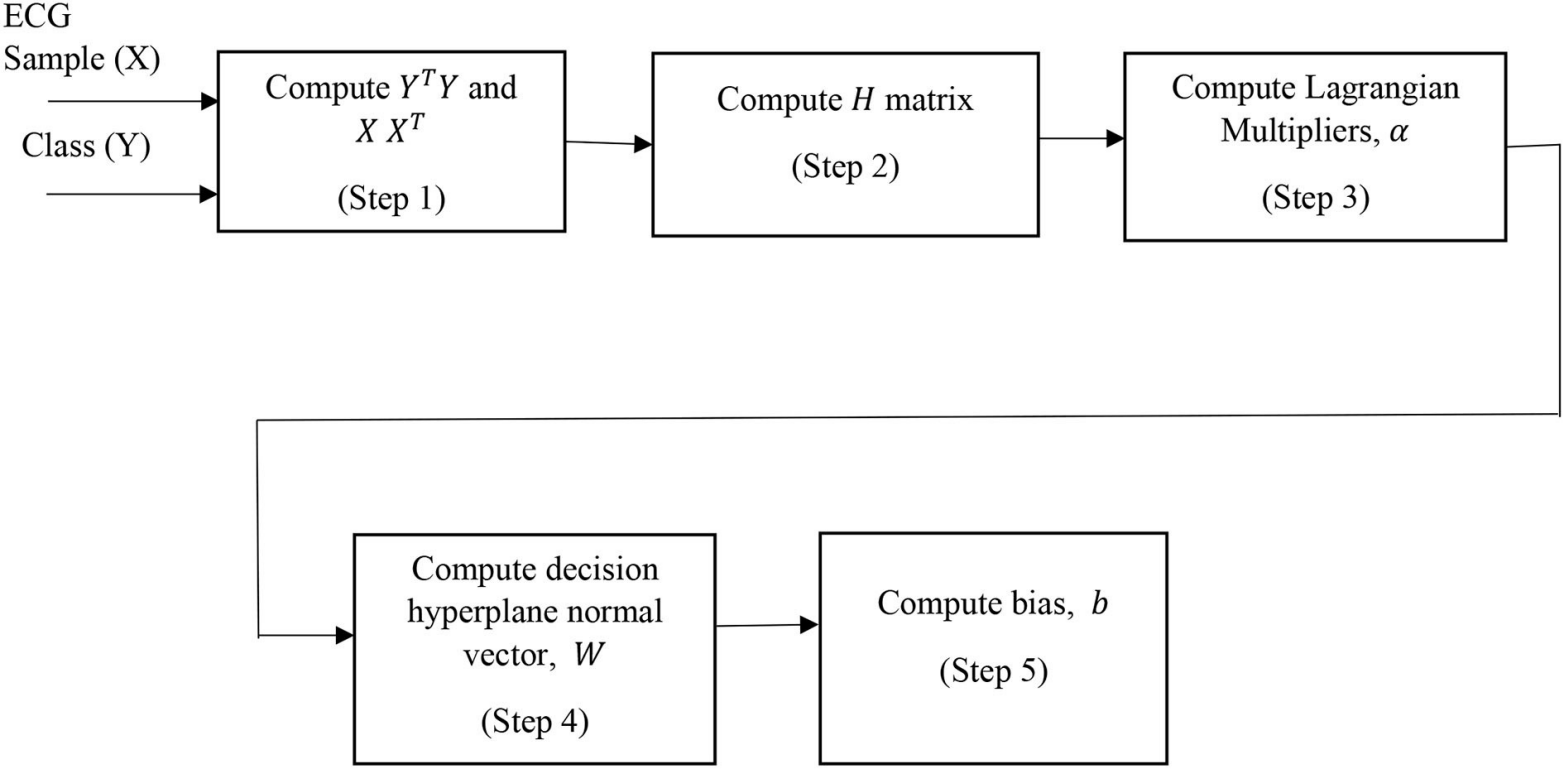
Block diagram of SVM classification model.

**Figure 3:**
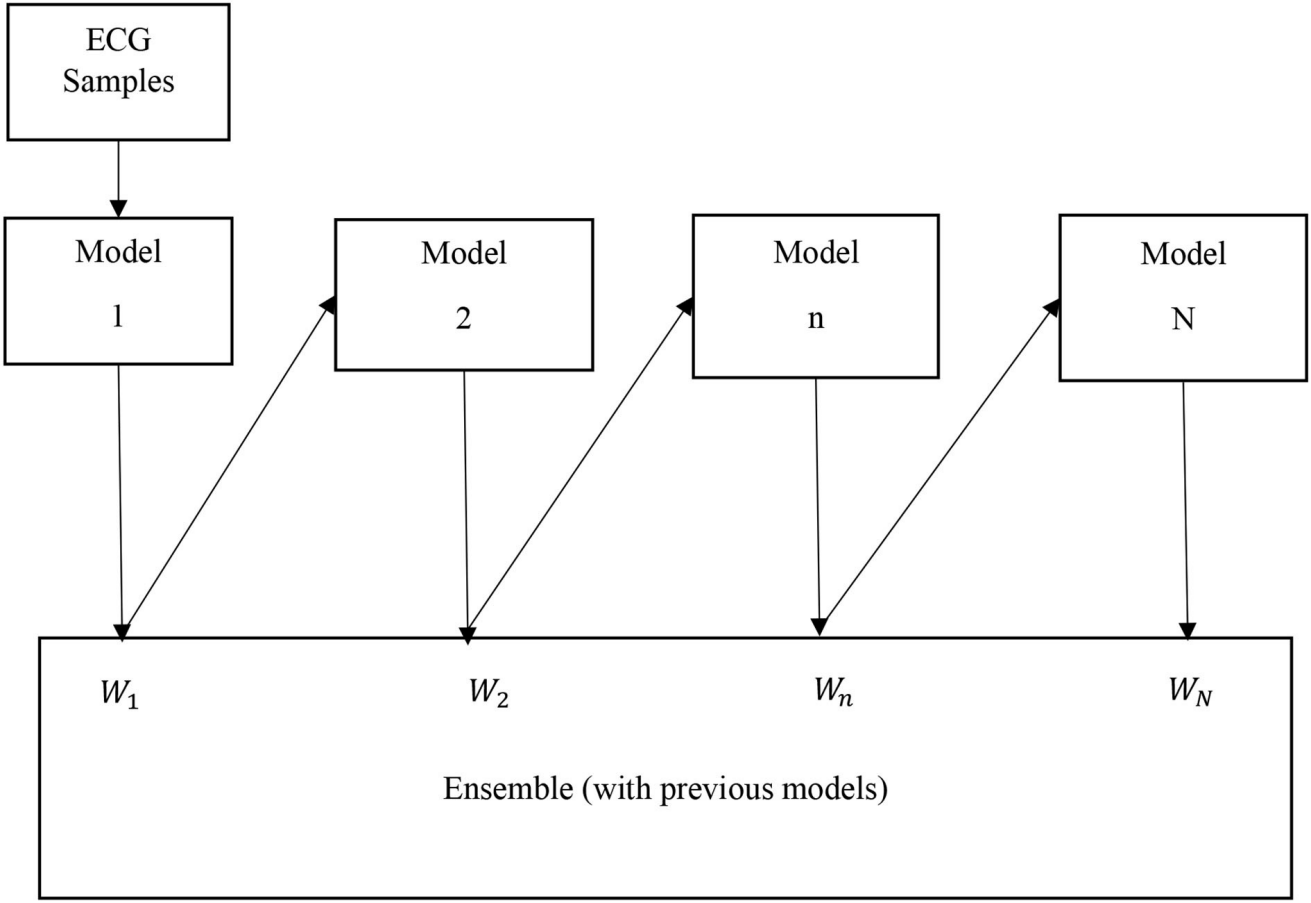
Block diagram of AdaBoost classification model.

**Figure 4:**
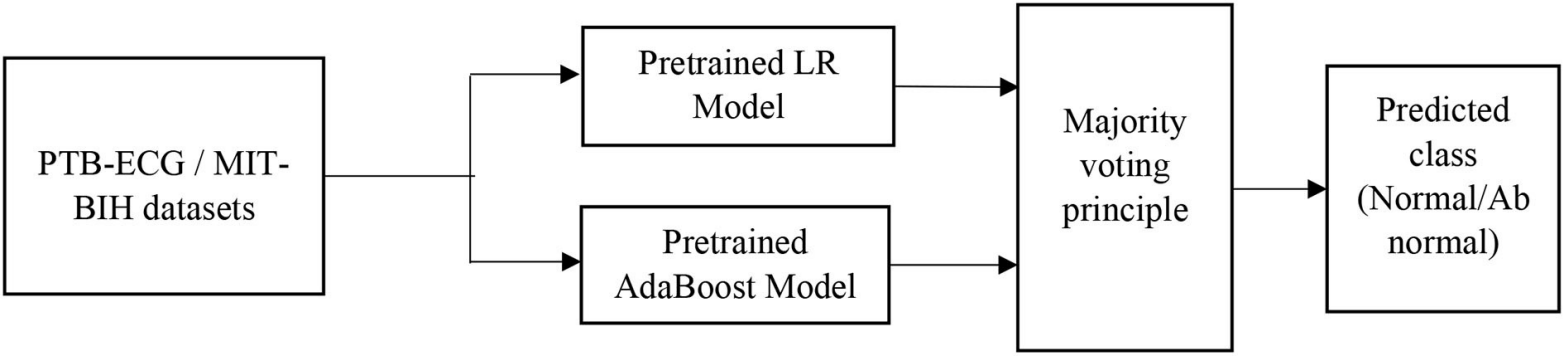
Block diagram of LR-AdaBoost ensemble model.

**Figure 5:**
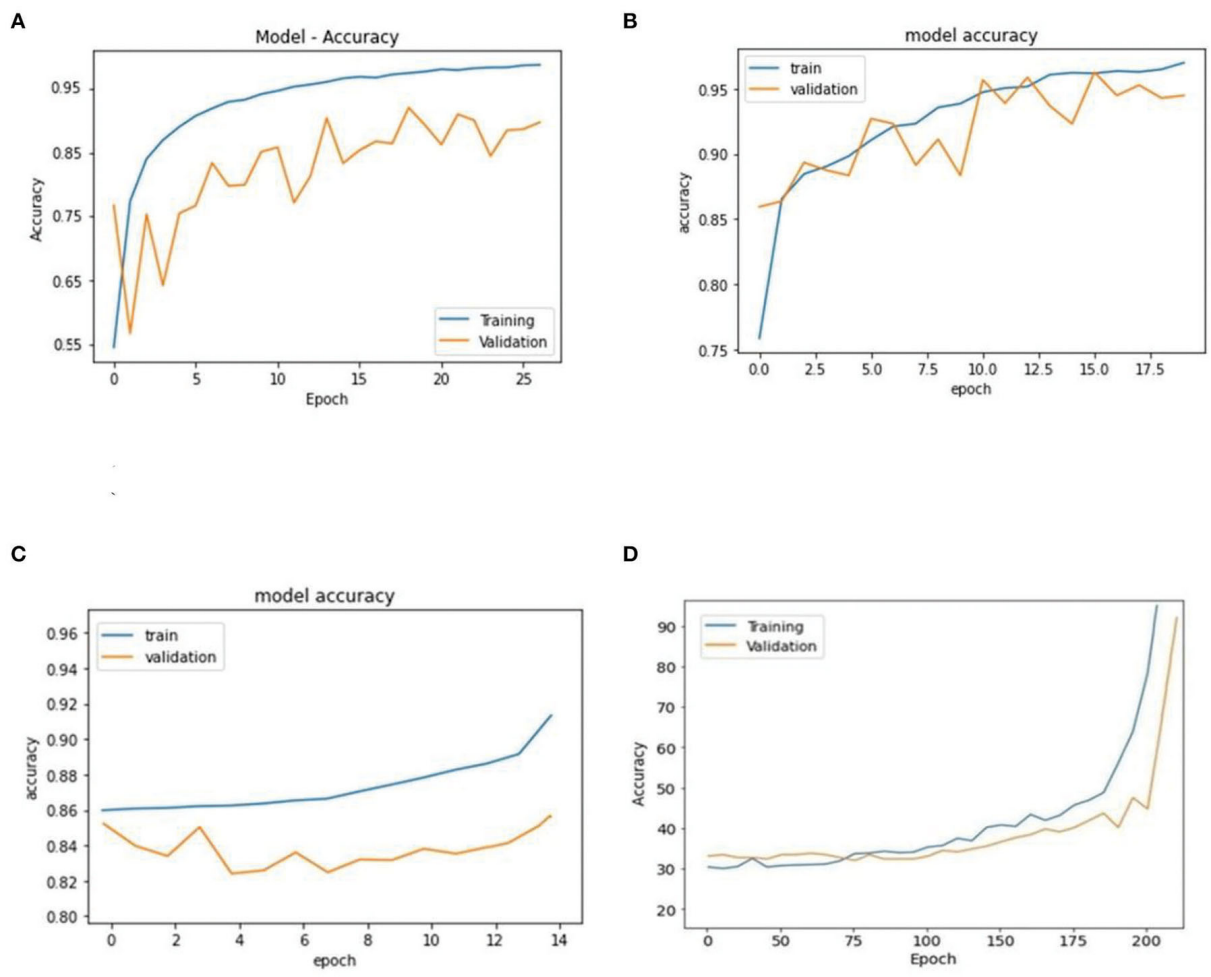
Comparison of accuracy achieved during training and validation phases of LR, AdaBoost, SVM, and ensemble of AdaBoost and LR models (PTB-ECG dataset). **(A)** Logistic regression. **(B)** AdaBoost. **(C)** Support vector machine (SVM). **(D)** AdaBoost – logistic regression based ensemble model.

**Figure 6:**
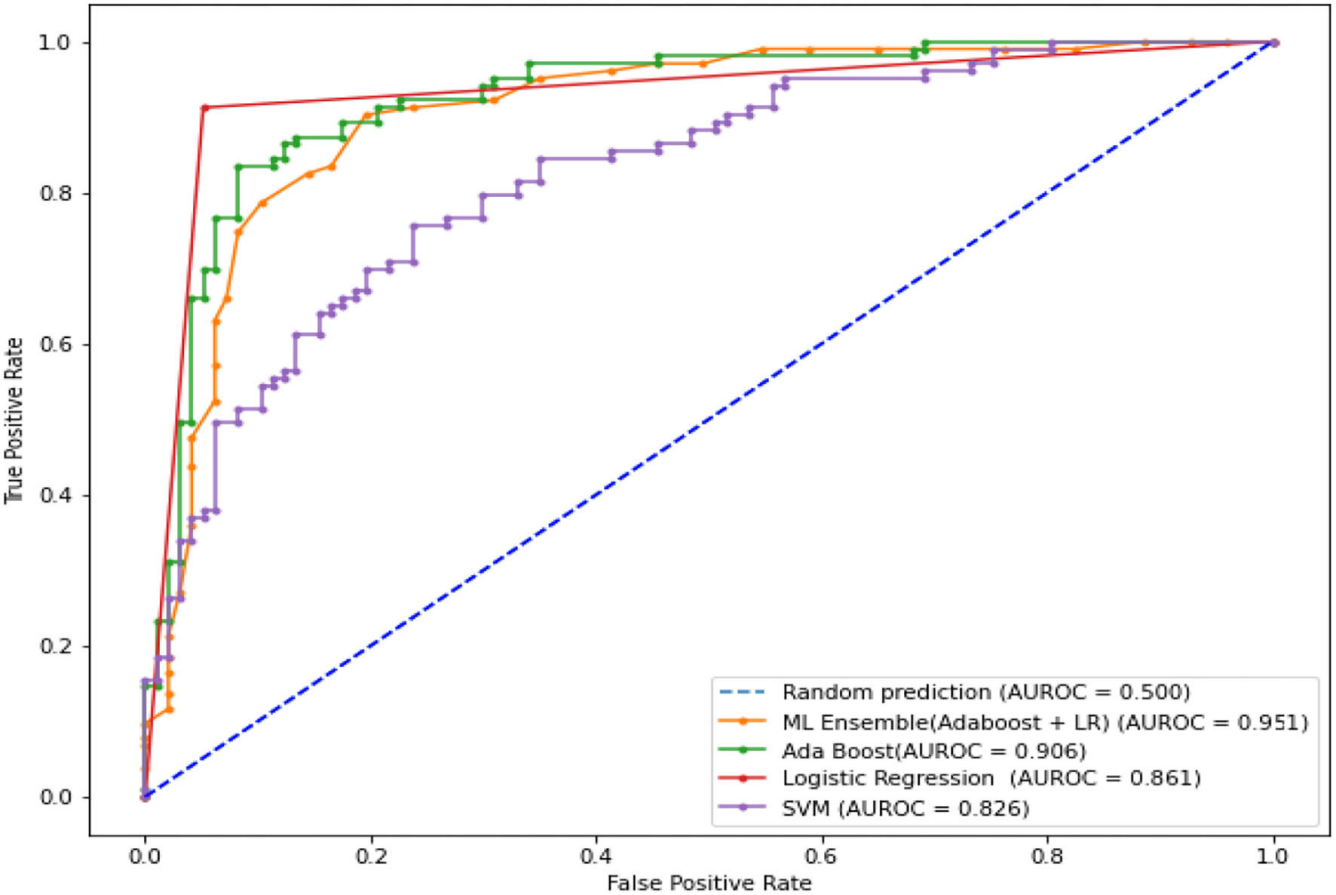
Comparison of ROC plots and AUC values of LR, AdaBoost, SVM, and LR-AdaBoost ensemble models (PTB-ECG dataset).

The LR, SVM, and AdaBoost classification models shown in Figures 1, 2, 3 have been simulated following the training principle of each of the model. Separate models have been simulated for each imbalanced PTB-ECG and MIT-BIH datasets as inputs. Similarly, the ensemble model shown in Figure 4 has been simulated using the same inputs. Each ECG record provides 1,024 samples which are simultaneously fed to the model both during the training and testing phases. In case of LR, the sigmoid function is used to keep the output between 0 and 1. In the simulation study, the learning rate alpha has been chosen to be 0.1. In case of AdaBoost, the decision tree has been used as the base estimator. In the present case, 30 decision trees have been used in the simulation study. In this case, the learning rate has been taken as 0.05. In case of the SVM classifier, the linear kernel has been used. For the PTB-ECG dataset, the plots of variation in accuracy with change in epochs during training and validation phases for LR, AdaBoost, SVM, and ensemble model are shown in Figures 5A–D, respectively. Further, the comparison of ROC plots obtained by LR, AdaBoost, SVM, and LR-AdaBoost ensemble model for the PTB-ECG dataset is shown in Figure 6. The same figure also provides the AUC values of these models. During the validation phase, the accuracy, F1-score, and AUC values of LR, SVM, AdaBoost, and ensemble (LR-AdaBoost) model have been determined and listed in Tables 2, 3 for PTB-ECG, and MIT-BIH datasets, respectively. The various results shown in the Tables and plotted in the graphs have been analyzed in the next section.

**Table 2:** Comparison of three performance metrics of different models using the PTB-ECG dataset.

**Performance measures**	**Logistic regression**	**SVM**	**AdaBoost**	**Ensemble model** **LR - AdaBoost**
Accuracy	0.898 (III)	0.864 (IV)	0.927 (II)	0.946 (I)
F1 Score	0.902 (III)	0.852 (IV)	0.936 (II)	0.949 (I)
AUC	0.861 (III)	0.826 (IV)	0.906 (II)	0.951 (I)

**Table 3:** Comparison of three performance metrics of different models using the MIT-BIH dataset.

**Performance measures**	**Logistic regression**	**SVM**	**AdaBoost**	**Ensemble model** **LR-AdaBoost**
Accuracy	0.869 (III)	0.821 (IV)	0.894 (II)	0.921 (I)
F1 Score	0.884 (III)	0.782 (IV)	0.845 (II)	0.926 (I)
AUC	0.858 (III)	0.819 (IV)	0.910 (II)	0.950 (I)

## Discussions

This section presents the interpretation of the various results presented in the previous sections. It is observed from the plots of Figure 5 that as the number of epochs increases, the accuracy value also increases and remains constant at the end of the training phase. Further, it is found that for any given epoch the training accuracy is higher than the corresponding validation accuracy. The observation of ROC plots of Figure 6 (PTB-ECG) dataset reveals that the ensemble model provides the highest AUC of 0.951. It is then followed by AdaBoost, LR, and SVM classification models. It is interesting to observe that the order in terms of magnitude of AUC values of different models is the same for both datasets as evident from Tables 2, 3. The observation of three important performance metrics (Accuracy, F1-score, AUC values) obtained from the simulation study of LR, SVM, AdaBoost, and ensemble model is shown in Table 2 (PTB-ECG dataset) and in Table 3 (MIT-BIH dataset). The observation shows that the ensemble model outperforms the individual three ML models. The bracketed terms such as (I), (II), etc. in Tables 2, 3 indicate the rank of respective classification models which are assigned based on the performance metrics. This is also evidenced by the individual and overall ranking assigned to these models in Tables 2, 3. In general, it is found that based on the three-performance metrics of all the four classification models and by employing imbalanced ECG data samples from two standard datasets, the rankings assigned are I, II, III, and IV for ensemble, AdaBoost, LR, and SVM models, respectively.

Based on the above analysis, the major contributions of investigation on HD detection are the following:

i. All the proposed classification models for HD detection using two imbalanced ECG recordings as subjects exhibit consistent performance following the imbalanced number of inputs both during the training and testing phases.
ii. As expected, the ensemble model developed using LR-AdaBoost has demonstrated the best performance among all the four models yielding accuracy, F1-score, and AUC values of 0.946, 0.949, and 0951 for the PTB-ECG dataset and 0.921, 0.926, and 0.950 for MIT-BIH dataset.
iii. These four models show similar performance for both the datasets as well as the following imbalanced number of ECG records as inputs to training and validation phases.

## Conclusion

This article has investigated the classification potentiality of HD using three ML algorithms and one ensemble model. The development of these models is based on imbalanced training ECG records. The accuracy plots and three performance measures reveal that the AdaBoost performs better than the SVM, LR-based classification models. This observation is true for both datasets. The LR-AdaBoost ensemble model based on majority voting principle demonstrates the best performance in terms of accuracy, F1-score, and AUC values compared to individual models. The numerical performance results also show that the order of the performance is consistent for both datasets. The present methodology can also be applied for HD detection using ICG, MCG, and HS signals. The HD detection results obtained from these three types of signals as inputs can be analyzed and compared with the results obtained from the present study. There are different kinds of ensemble techniques that can be employed for developing the ensemble model. The results of these ensemble models can be compared based on the performance and the best model can be chosen. The proposed approaches can also be applied to the detection of other diseases.

## Data availability statement

Publicly available datasets were analyzed in this study. This data can be found here: MIT-BIH ECG datasets - https://physionet.org/content/mitdb/1.0.0/ and PTB-ECG datasets - https://www.physionet.org/content/ptbdb/1.0.0/.

## Author contributions

All authors listed have made a substantial, direct, and intellectual contribution to the work and approved it for publication.

## Conflict of interest

The authors declare that the research was conducted in the absence of any commercial or financial relationships that could be construed as a potential conflict of interest.

## Publisher's note

All claims expressed in this article are solely those of the authors and do not necessarily represent those of their affiliated organizations, or those of the publisher, the editors and the reviewers. Any product that may be evaluated in this article, or claim that may be made by its manufacturer, is not guaranteed or endorsed by the publisher.
